# Low-dose radiation exposure and risk of self-reported cataract in Fukushima nuclear emergency workers

**DOI:** 10.1093/ije/dyag063

**Published:** 2026-05-20

**Authors:** Huan Hu, Natsuko Hatsusaka, Mark P Little, Osamu Kurihara, Eunjoo Kim, Hiroshi Sasaki, Tetsuya Mizoue, Toshiteru Okubo

**Affiliations:** Research Center for Prevention from Radiation Hazards of Workers, National Institute of Occupational Safety and Health, Kanagawa 214-8585, Japan; Department of Ophthalmology, Kanazawa Medical University, Ishikawa 920-0293, Japan; Faculty of Health, Science and Technology, Oxford Brookes University, Oxford OX3 0BP, United Kingdom; Feinberg School of Medicine, Northwestern University, Evanston, IL 60208, United States; Department of Radiation Measurement and Dose Assessment, Institute of Radiological Sciences, National Institutes for Quantum Science and Technology, Chiba 263-8555, Japan; Department of Radiation Measurement and Dose Assessment, Institute of Radiological Sciences, National Institutes for Quantum Science and Technology, Chiba 263-8555, Japan; Department of Ophthalmology, Kanazawa Medical University, Ishikawa 920-0293, Japan; Research Center for Prevention from Radiation Hazards of Workers, National Institute of Occupational Safety and Health, Kanagawa 214-8585, Japan; Department of Epidemiology and Prevention, Center for Clinical Sciences, Japan Institute of Health Security, Tokyo 162-8655, Japan; Research Center for Prevention from Radiation Hazards of Workers, National Institute of Occupational Safety and Health, Kanagawa 214-8585, Japan

**Keywords:** low-dose radiation, cataract risk, Fukushima Daiichi nuclear accident, nuclear emergency workers

## Abstract

**Background:**

Cataract is a well-established radiogenic condition and recent evidence suggests an increased risk even at radiation doses of <100 mGy. Following the 2011 Fukushima Daiichi Nuclear Power Plant accident, ∼20 000 workers participated in emergency operations. This study aimed to investigate the association between cumulative low-dose occupational radiation exposure and the risk of cataract among workers enrolled in the Epidemiological Study of Health Effects in Fukushima Nuclear Emergency Workers.

**Methods:**

Data from 5773 nuclear emergency workers participating in the cohort were analysed. The cumulative lifetime occupational radiation dose was estimated by combining pre-2011 individual dose records from a nationwide nuclear radiation worker dose registry with radiation doses received during the 2011 emergency-work period. Self-reported, physician-diagnosed cataract was identified through baseline and first follow-up questionnaires. A Cox proportional hazards model was applied to estimate hazard ratios (HRs) with 95% confidence intervals (CIs) for incident cataract, with adjustments for major covariates.

**Results:**

More than 90% of the participants had cumulative occupational radiation exposure of <100 mSv, including doses received both prior to the accident and during the emergency period (March–November 2011). During 52 921 person-years of follow-up from 2012 to 2024 (mean, 9.2 years), 310 participants reported new-onset cataract. The risk of cataract increased with lifetime occupational exposure up to November 2011, with an HR of 1.03 per 10-mSv increase (95% CI, 1.02, 1.05). This association persisted after excluding participants with doses of ≥100 mSv. For exposure during the emergency period (March–November 2011), the HR was 1.06 per 10-mSv increase (95% CI, 1.03, 1.09). When the cumulative lifetime occupational radiation dose was recalculated by using 2-year and 5-year lag periods before diagnosis or censoring, the HRs per 10-mSv increase were 1.03 (95% CI, 1.01, 1.04) and 1.01 (95% CI, 0.99, 1.04), respectively.

**Conclusion:**

This study suggests an excess risk of cataract at doses of <100 mSv and adds to the evidence base suggesting an association between low-dose occupational radiation exposure and cataract risk.

Key Messages

- We investigated whether low-dose occupational radiation exposure was associated with cataract risk among Fukushima nuclear emergency workers.
- We observed a dose–response relationship, with cataract risk increasing at Hp(10) doses of <100 mSv.
- These findings add to the evidence base suggesting an association between low-dose occupational radiation exposure and cataract risk, but should be interpreted with caution given the self-reported cataract status and other lifestyle and medical data, dosimetry, and other limitations of the study.

## Background

Cataract is a common age-related condition worldwide and a leading cause of visual impairment [1]. In Japan, the estimated prevalence of clinically diagnosed cataract was ∼7% in 2020 based on data from a commercial administrative claims database [2]. Although age is the primary risk factor, accumulating evidence indicates that radiation exposure may also contribute to cataract development. High-dose ionizing radiation (≥1 Gy) is widely recognized as a cause of cataract [3]. In addition, studies of diverse radiation-exposed populations, including atomic-bomb survivors [4], Chernobyl cleanup workers [5], Mayak nuclear workers [6], and residents of areas with high natural background radiation [7], have reported increased risks of cataract at moderate dose levels (0.1–1.0 Gy). However, the effects of low-dose radiation (<100 mGy) remain unclear. To date, the United States Radiologic Technologists (USRT) study is the only large-scale cohort investigation to have reported an association between low-dose radiation exposure (<100 mGy) and the risk of cataract [8, 9].

The Fukushima Daiichi Nuclear Power Plant accident, classified as Level 7 (Major Accident) on the International Nuclear Event Scale, involved ∼20 000 workers who performed emergency tasks between 14 March and 16 December 2011. Most of these workers received radiation doses of <100 mSv (approximately equivalent to 100 mGy for γ- and x-rays) during the emergency work [10]. In response to concerns about the potential long-term health effects of this exposure, the Epidemiological Study of Health Effects in Fukushima Nuclear Emergency Workers (NEWS) was initiated in 2014 to investigate these risks [11]. In the present study, the association between low-dose radiation exposure and the risk of cataract was examined using data from the NEWS cohort.

## Methods

In this prospective analysis, data from the NEWS study—an ongoing cohort of workers who performed emergency tasks following the 2011 Fukushima Daiichi Nuclear Power Plant accident—were used. Details of the NEWS study have been published elsewhere [11]. Briefly, the baseline (first) survey (conducted between 2016 and 2021) included comprehensive physical and laboratory examinations and a questionnaire collecting information on lifestyle, medical history, radiation-related emergency-work details, and other relevant factors. A second comprehensive survey was conducted 4–5 years after the baseline survey. Ethical approval was obtained from the ethics committees of the Radiation Effects Research Foundation (approval no. RP 6–15) and the National Institute of Occupational Safety and Health (approval no. 2024N28). All participants provided informed consent.

## Participants

Of the 19 812 emergency workers registered in the Ministry of Health, Labour and Welfare (MHLW) database, ∼19 700 were invited to participate in the NEWS study by mail, beginning in August 2015. Between 2016 and 2021, 6087 workers completed the baseline survey. Women were excluded from the present analysis because of their limited numbers (*n* = 10). Further exclusions were participants with a history of physician-diagnosed cataract prior to 2012 (*n* = 90), participants with missing information on occupational radiation exposure prior to 2011 (*n* = 207), and participants with incomplete covariate information (*n* = 7). After these exclusions, the final analytic cohort consisted of 5773 participants. Of these, 4231 completed the second survey between 2020 and 2024.

## Ascertainment of cataract

Self-reported, physician-diagnosed cataract was ascertained from both the first and second questionnaire surveys. Participants were asked to report the age at onset and to select one of the following treatment statuses: untreated; under observation; under treatment; post-surgery under treatment; post-surgery cured; or cured (surgery status unspecified). New-onset cataract cases occurring between 2012 and 2024 were identified when the reported age at onset exceeded the participant’s age in 2011.

## Exposure assessment

### Radiation-dose assessment during the 2011 emergency-work period

During the emergency-work period (March to December 2011), each emergency worker was issued a personal pocket alarm dosimeter before entering the workplace each day. The dosimeters were returned to a designated gatekeeper at the end of each workday and a qualified specialist recorded the daily accumulated dose in the Tokyo Electric Power Company dose-management database. However, from 15 to 31 March 2011, group dose estimates were used for some workers because of a shortage of individual pocket dosimeters. For workers potentially exposed to internal radiation, internal doses were measured by using whole-body counters at appropriate intervals. All radiation-dose records were subsequently transferred to the MHLW database. For this study, radiation-dose data were provided by the MHLW. Radiation doses received during the emergency-work period (March–November 2011) were calculated as the sum of the external and internal doses. Doses received in December 2011 were excluded because they were considered minimal and were available only as monthly totals.

### Cumulative lifetime occupational exposure through November 2011

Occupational radiation-dose data were obtained from the Radiation Effects Association (REA), which has maintained the centralized dose registry for all nuclear power plant workers in Japan since 1977 [12, 13]. The REA provided annual individual doses, expressed in millisieverts, comprising both external and internal components. External doses corresponded to the measured personal dose equivalents, H_p_(10), from penetrating radiation (i.e. photons and neutrons), whereas internal doses were the committed effective doses assessed by using direct *in vivo* measurements. Occupational radiation exposure was predominantly external, with internal exposure rarely detected, even during emergency operations. The cumulative lifetime occupational exposure through November 2011, the primary exposure metric in this study, was calculated by combining pre-2011 dose records from the REA with radiation-dose assessments from the 2011 emergency-work period. Dose data after 2011 were used in sensitivity analyses incorporating lagged cumulative-dose calculations.

### Covariates

Covariates included age (as of 31 December 2011), employment status (whether the participant was employed by the Tokyo Electric Power Company during the 2011 emergency-work period), body mass index (BMI), alcohol consumption, smoking status, and diabetes status, which were obtained from the 2016–21 baseline survey. These variables were selected because they represent known risk factors for cataract [14–16] and have been applied for background rate adjustment in previous studies [5, 6, 9]. In the baseline survey, body weight and height were measured by using a scale, with participants wearing light clothes and no shoes. BMI was calculated as weight in kilograms divided by height in meters squared. Alcohol consumption (current or non-current drinker) and smoking status (never, former, or current smoker) were obtained through a self-administered questionnaire. Diabetes was defined as glycated hemoglobin (HbA1c) of ≥6.5%, fasting glucose of ≥126 mg/dL, a self-reported physician diagnosis, or the use of anti-diabetic medication.

### Statistical analysis

The characteristics of the study participants were summarized as means (continuous variables) and percentages (categorical variables). Participants were classified into exposure groups based on their cumulative radiation doses: 0–4, 5–9, 10–19, 20–49, 50–99, and ≥100 mSv, consistently with the cutoff points used in previous studies [8, 17, 18]. Kaplan–Meier curves were used to describe the time to incident cataract across groups of cumulative lifetime occupational radiation exposure. Person-time was calculated from 31 December 2011 to either the estimated date of cataract onset (defined as the reported age at onset from the questionnaire plus 0.5 years minus the participant’s age on 31 December 2011) or to the date of the most recent survey (baseline or second), whichever occurred first.

The association between cataract risk and two measures of radiation exposure was examined: exposure received during the emergency-work period (March–November 2011) and cumulative lifetime occupational exposure up to November 2011, with the latter used as the main exposure. Cox proportional hazards regression was used to estimate the hazard ratios (HRs) and 95% confidence intervals (CIs) for cataract risk associated with radiation exposure, adjusting for age, employment status, BMI, smoking status, and diabetes. Interactions between radiation exposure and covariates were tested by including interaction terms in the Cox proportional hazards regression models.

Several sensitivity analyses were conducted to assess the robustness of the findings. First, early-stage cases (those untreated or under observation) were excluded to mitigate potential detection bias, because individuals with higher radiation exposure may have undergone more frequent medical examinations. Second, to account for the potential latency of radiation-induced cataract, cumulative doses were lagged by 2 and 5 years, respectively. For example, if a participant reported a cataract diagnosis in 2018, the lifetime cumulative dose was calculated through 2015 or 2012, respectively, reflecting the availability of annual (rather than monthly) dose records. All statistical analyses were performed by using SAS version 9.4 (SAS Institute, Cary, NC, USA).

## Results

Of the 5773 participants included in the analysis, the mean age in 2011 was 46.5 years (SD 10.4) and the mean BMI was 24.4 (3.5) kg/m^2^; 33.9% were current smokers and 11.5% had diabetes. More than 90% of the participants had cumulative lifetime occupational radiation exposure of <100 mSv as of November 2011; the mean dose was 26.1 mSv (SD 46.5), with an interquartile range of 1.2–29.5 mSv. As shown in Table 1, these characteristics did not differ substantially across the dose groups, except that participants in the higher-dose groups were more likely to have had occupational radiation exposure before 2011 and to be employees of the Tokyo Electric Power Company.

**Table 1:** Characteristics of study participants.

	Cumulative lifetime occupational exposure up to November 2011 (mSv)						Total
	0–4	5–9	10–19	20–49	50–99	≥100	
*N*	2487	645	795	915	551	380	5773
Age in 2011 (years) (mean ± SD)	47.0 ± 10.3	45.2 ± 10.4	44.9 ± 10.8	46.2 ± 10.0	46.4 ± 10.5	49.9 ± 9.5	46.5 ± 10.4
BMI (kg/m^2^) (mean ± SD)	24.4 ± 3.4	24.6 ± 3.7	24.6 ± 3.7	24.3 ± 3.3	24.5 ± 3.9	25.0 ± 4.3	24.4 ± 3.5
Current drinker (%)	84.2	86.4	82.8	83.0	84.8	85.4	84.0
Current smoker (%)	32.2	34.4	35.9	37.3	32.1	34.2	33.9
Diabetes (%)	10.4	9.2	13.6	13.1	10.9	15.0	11.5
Participants with occupational radiation exposure before 2011 (%)	15.3	36.4	50.3	66.0	90.2	99.0	43.2
Employment at Tokyo Electric Power Company in 2011 (%)	34.0	34.6	41.6	40.3	51.2	40.5	38.2

**Figure 1:**
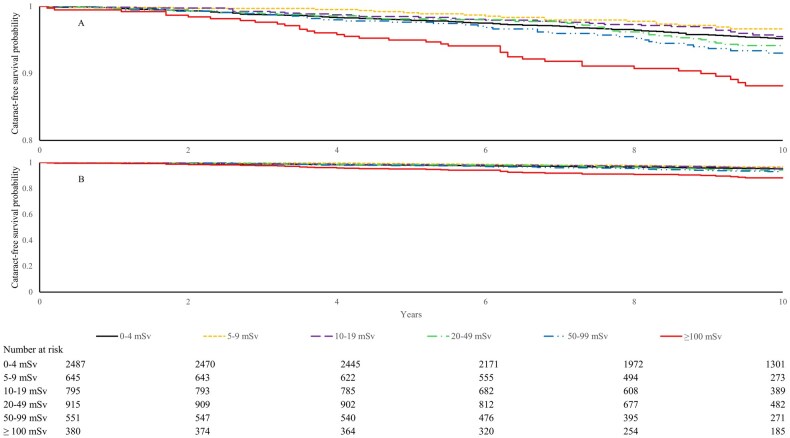
Kaplan–Meier curves for cataract-free survival by cumulative lifetime radiation exposure groups. (A) Cataract-free survival over the 10-year follow-up period, with a restricted *y*-axis scale (0.8–1.0) to highlight differences between the exposure groups; (B) the same data over the same follow-up period, displayed using the full *y*-axis scale (0–1.0). The lines represent cumulative radiation-dose categories of 0–4, 5–9, 10–19, 20–49, 50–99, and ≥100 mSv, respectively.

Between 2012 and 2024, 310 participants reported physician-diagnosed new-onset cataract during 52 921 person-years of follow-up (mean follow-up, 9.2 years; range, 0.1–13.1 years). Among these cases, 11.3% were untreated, 26.5% were under observation, 18.7% were under treatment, 12.9% were post-surgery under treatment, and 30.6% were reported as cured (29.0% post-surgery cured; 1.6% cured with surgery status unspecified). Kaplan–Meier curves showed differences in cataract risk across the cumulative lifetime occupational radiation exposure groups, with lower cataract-free probabilities observed in the 50–99 and ≥100 mSv groups (Fig. 1).

Table 2 presents the HRs for cataract by radiation-dose groups. The risk of cataract increased with cumulative lifetime occupational radiation exposure up to November 2011. Compared with the 0–4 mSv group, the multivariable-adjusted HRs were 0.89 (95% CI, 0.57, 1.40) for 5–9 mSv, 1.13 (95% CI, 0.78, 1.63) for 10–19 mSv, 1.24 (95% CI, 0.89, 1.73) for 20–49 mSv, 1.49 (95% CI, 1.02, 2.16) for 50–99 mSv, and 2.07 (95% CI, 1.46, 2.95) for ≥100 mSv. When the cumulative radiation dose was analysed as a continuous variable, the HR per 10-mSv increase was 1.03 (95% CI, 1.02, 1.05). Among participants with cumulative doses of <100 mSv, the HR per 10-mSv increase was 1.06 (95% CI, 1.01, 1.11). For radiation exposure during the emergency-work period, the corresponding HRs were 1.07 (95% CI, 0.74, 1.54), 1.24 (95% CI, 0.88, 1.74), 1.37 (95% CI, 0.99, 1.90), 2.86 (95% CI, 1.89, 4.32), and 2.00 (95% CI, 0.87, 4.59), respectively. When analysed as a continuous variable, the HR per 10-mSv increase was 1.06 (95% CI, 1.03, 1.09). Among participants with radiation doses of <100 mSv during the emergency-work period, the HR per 10-mSv increase was 1.15 (95% CI, 1.08, 1.21). The results for radiation exposure during the emergency-work period remained essentially unchanged after further adjustment for cumulative occupational radiation exposure up to February 2011. Estimates for all covariates are provided in Supplementary Table 1. No evidence of an interaction between radiation exposure and the covariates was observed (Supplementary Table 2).

**Table 2:** Radiation exposure and risk of cataract.

	Radiation exposure (mSv)						Per 10 mSv	*P* for trend
	0–4	5–9	10–19	20–49	50–99	≥100		
Radiation exposure received during the emergency-work period								
Median dose	0.80	7.33	13.80	30.10	65.27	125.69		
Cases/*N*	149/2978	35/751	43/856	49/817	28/272	6/99	310/5773	
HR (95% CI)^a^	Reference	1.07 (0.74, 1.54)	1.24 (0.88, 1.74)	1.37 (0.99, 1.90)	2.86 (1.89, 4.32)	2.00 (0.87, 4.59)	1.06 (1.03, 1.09)	<.001
HR (95% CI)^b^	Reference	1.04 (0.72, 1.50)	1.18 (0.84, 1.67)	1.29 (0.93, 1.80)	2.64 (1.73, 4.02)	1.72 (0.74, 3.99)	1.06 (1.03, 1.09)	<.001
Cumulative lifetime occupational exposure up to November 2011								
Median dose	0.86	7.18	14.08	31.40	67.50	145.05		
Cases/*N*	122/2487	23/645	37/795	50/915	36/551	42/380	310/5773	
HR (95% CI)^a^	Reference	0.89 (0.57, 1.40)	1.13 (0.78, 1.63)	1.24 (0.89, 1.73)	1.49 (1.02, 2.16)	2.07 (1.46, 2.95)	1.03 (1.02, 1.05)	<.001

a Adjusted for age, employment at Tokyo Electric Power Company, BMI, alcohol consumption, smoking status, and diabetes.

b Adjusted for age, employment at Tokyo Electric Power Company, BMI, alcohol consumption, smoking, diabetes, and cumulative radiation exposure up to February 2011.

As shown in Table 3, the results for cumulative lifetime occupational exposure up to November 2011 remained materially unchanged after the exclusion of cases that were untreated or under observation, with an HR of 1.03 (95% CI, 1.00, 1.05) per 10-mSv increase. When the cumulative lifetime occupational radiation dose was calculated using 2-year and 5-year lag periods, the HRs per 10-mSv increase were 1.03 (95% CI, 1.01, 1.04) and 1.01 (95% CI, 0.99, 1.04), respectively.

**Table 3:** Cumulative lifetime occupational radiation exposure and risk of cataract, excluding untreated cases and applying exposure lag periods.

	Cumulative lifetime occupational exposure (mSv)						Per 10 mSv	*P* for trend
	0–4	5–9	10–19	20–49	50–99	≥100		
Excluded cases: untreated or under observation^a^								
Median dose	0.87	7.19	14.06	31.41	67.59	144.08		
Cases/*N*	83/2448	14/636	25/783	29/894	19/534	23/361	193/5656	
HR (95% CI)	Reference	0.85 (0.48, 1.49)	1.16 (0.74, 1.82)	1.10 (0.72, 1.69)	1.24 (0.75, 2.05)	1.72 (1.08, 2.73)	1.03 (1.00, 1.05)	.02
2-year lag^b^								
Median dose	0.86	7.08	14.20	33.09	67.58	137.01		
Cases/*N*	126/2480	29/653	39/674	42/998	39/628	35/340	310/5773	
HR (95% CI)	Reference	1.05 (0.70, 1.58)	1.29 (0.90, 1.85)	0.94 (0.66, 1.33)	1.33 (0.93, 1.91)	1.73 (1.19, 2.53)	1.03 (1.01, 1.04)	.01
5-year lag^b^								
Median dose	0.68	7.01	14.12	33.35	67.20	134.74		
Cases/*N*	155/2666	23/629	30/630	38/987	33/563	31/298	310/5773	
HR (95% CI)	Reference	0.71 (0.46, 1.11)	0.86 (0.58, 1.28)	0.70 (0.49, 1.01)	0.97 (0.66, 1.42)	1.38 (0.93, 2.03)	1.01 (0.99, 1.04)	.18

a Adjusted for age, employment at Tokyo Electric Power Company, BMI, alcohol consumption, smoking, and diabetes.

b Age was used as the timescale for the Cox proportional hazards model, defined by two points: age at the end of 2011 (entry into follow-up) and attained age at the time of the event or censoring.

## Discussion

In this cohort study of Fukushima nuclear emergency workers, a linear association was observed between cumulative lifetime occupational radiation exposure and the risk of self-reported, physician-diagnosed cataract. The risk increased notably among individuals who received a cumulative dose of ≥50 mSv. This study is among the few to have investigated the association between low-dose occupational radiation exposure (<100 mSv) and cataract risk.

Although the International Commission on Radiological Protection reclassified cataract as a tissue reaction and recommended a threshold dose of 500 mGy below which no excess risk is expected [19], the findings of this study indicate an elevated cataract risk at Hp(10) doses of <100 mSv. These results are consistent with findings from the USRT cohort, which followed >60 000 radiologic technologists for a mean of 13 years and identified >12 000 cataract cases. An association was observed between self-reported cataract and absorbed doses to the eye lens of <100 mGy, despite at low mean dose of ∼50 mGy (interquartile range, 20–70 mGy) [8, 9, 20]. Similarly, studies of Mayak nuclear workers and residents of areas in China with high natural background radiation have reported elevated cataract risks at cumulative doses of between 100 and 500 mSv [6, 7]. Taken together with the results of previous studies, these findings add to the epidemiological evidence suggesting an association between low-dose radiation exposure and cataract risk.

Among the Fukushima nuclear emergency workers, individuals who received ≥50 mSv during emergency work may have undergone more frequent ophthalmologic examinations [10], potentially increasing the detection of early-stage cataract. Considering only the emergency period, the HRs for cataract were elevated in the 50–99 and ≥100 mSv groups (HR: 2.86 and 2.00, respectively). In contrast, when the cumulative lifetime exposure up to November 2011 was considered, the HRs were 1.49 and 2.07, respectively, suggesting possible detection bias. However, sensitivity analyses excluding early-stage cases (untreated or under observation) demonstrated that the elevated risk persisted among individuals exposed to >50 mSv. Furthermore, key confounders, including age, alcohol consumption, smoking status, and diabetes, were adjusted for in all analyses. Taken together, these findings suggest that detection bias alone is unlikely to account for the observed association and support a genuine dose–response relationship at low occupational radiation doses.

The mechanisms by which low-dose radiation contributes to cataract formation remain under investigation. Rodent studies have demonstrated that such exposure can induce micronucleus formation, the disorganization of meridional rows, and progressive lens opacification [21]. Furthermore, an *in vitro* study in which human lens epithelial cells were used suggested that low-dose radiation may promote cataractogenesis by causing DNA damage and by disrupting normal cell differentiation via transient p21 activation [22]. Another *in vitro* study reported low-dose radiation-induced oxidative stress, DNA damage, and cellular senescence in human lens epithelial cells, suggesting that multiple pathways may be involved in radiation-induced cataractogenesis [23]. There is evidence for a somatic mutational component in at least some human cataract [24, 25]. Although the underlying mechanisms remain uncertain, the potential involvement of somatic cell mutation makes it biologically plausible that cataract risk may increase at low radiation doses, in a manner analogous to cancer.

## Strengths and limitations

The strengths of the study include the use of personal dosimeter (badge) monitoring during the emergency-work period and the incorporation of registered occupational dose data prior to 2011. A large proportion of the participants (5393 of 5773; 93%) had low cumulative doses (<100 mSv), with nearly half (2487 of 5773; 43%) exposed to very low doses (<5 mSv). Established potential confounders such as age, BMI, smoking, alcohol consumption, and diabetes were adjusted for in the analysis. Results were robust to the exclusion of cases that were untreated or under observation (Table 3). As surgically treated cases, which are likely to be more severe, are much less likely to be subject to variations in ascertainment, this provides reassurance that variations in ascertainment may be limited. Sensitivity analyses were also employed to assess the effect of lagging dose. The use of a lag period of 2 years did not materially change the excess risks, whereas the use of a lag period of 5 years attenuated the excess risk (Table 3).

However, this study has some limitations. The dose used for the analyses was the personal dose equivalent, Hp(10), measured by using personal dosimeters. While this metric may reasonably reflect the external gamma-ray dose to the eye, depending on the exposure geometry, it does not account for potential internal radiation doses and specific data on actual lens doses were not available. Details about eye protection during the emergency-work period, as well as prior occupational radiation exposure among individuals with previous radiation-related jobs, were not collected, limiting our ability to evaluate any protective effects. The cataract status was based on self-reported physician diagnoses without medical validation, which may have caused inconsistent reporting, possibly influenced by the level of radiation exposure, or inaccurate recall of when cataract first developed. The frequency of eye examinations was not recorded. Information on cataract types was also not available in the questionnaire, limiting the assessment of biological specificity. The distinction between an opacity—a minor change not affecting sight—and a cataract would also be crucial. The large fraction of the reported cases being untreated or merely under observation (with fewer than half operated on) suggests that a substantial proportion of the cases are not frank cataract. In addition, misclassification of other eye diseases as cataract cannot be entirely excluded, although such misclassification is likely to be limited. Although key confounders were adjusted for, residual confounding due to unmeasured factors, including ultraviolet exposure, corticosteroid use, and ocular trauma, cannot be excluded. Although genetic predisposition and inflammation are important risk factors, they are unlikely to be correlated with radiation exposure and are therefore less likely to confound the observed associations. Selection bias is a potential concern, as respondents may differ systematically from nonrespondents with respect to health status or radiation exposure. This concern is heightened by the relatively low response rate to the initial invitation to participate (6087 of 19 812; 31%). The duration of follow-up was relatively short, at ∼10 years. However, cataract are known to occur relatively soon after radiation exposure; for example, among the Japanese atomic-bomb survivors, radiation-associated cataract was observed within ∼2.5 years of the bombings [26]. If the preferential ascertainment of cataract occurred among workers with higher radiation doses, then the associated risks would likely have been overestimated. To explore this possibility, we conducted analyses restricted to treated cataract cases, which are less susceptible to detection bias. The similarity of risk estimates in these analyses suggests that differential surveillance alone is unlikely to fully explain the observed associations.

## Conclusion

In conclusion, a linear association was observed between low-dose radiation exposure and the risk of self-reported cataract among Fukushima nuclear emergency workers. Nevertheless, these findings are based on limited follow-up and are subject to the limitations discussed above. Further follow-up of this cohort is crucial to confirm these findings and to provide deeper insights into the long-term effects of low-dose radiation exposure.

## Ethics approval

The study protocol was approved by the ethics committees of the Radiation Effects Research Foundation (approval no. RP 6–15) and the National Institute of Occupational Safety and Health (approval no. 2024N28).

## Supplementary Material

dyag063_Supplementary_Data

## Data Availability

The data underlying this article cannot be shared publicly due to privacy and ethical restrictions.
