# Supplementary material for: Low-dose radiation exposure and risk of self-reported cataract in Fukushima nuclear emergency workers
Source: Int J Epidemiol. 2026 May 20;55(3):dyag063. doi: 10.1093/ije/dyag063 (PMC13188973; doi:10.1093/ije/dyag063)
Supplement: dyag063_Supplementary_Data [file dyag063_supplementary_data.docx]

**Supplementary material**

Supplementary Table S1 Association between occupational radiation exposure and cataract risk, adjusted for selected covariates

| **Covariates** | N | Hazard ratio (95% CI) | P-value |
| --- | --- | --- | --- |
| **Cumulative lifetime occupational exposure up to November 2011 (per 10 mSv)** | 5773 | 1.03 (1.02, 1.05) | <0.001 |
| **Age in 2011 (years)** | 5773 | 1.10 (1.08, 1.12) | <0.001 |
| **Body mass index (kg/m^2^)** | 5773 | 0.99 (0.96, 1.03) | 0.70 |
| **Current smokers** |  |  |  |
| No | 3817 | Reference |  |
| Yes | 1956 | 0.81 (0.62, 1.08) | 0.16 |
| **Curren drinkers** |  |  |  |
| No | 924 | Reference |  |
| Yes | 4849 | 0.82 (0.62, 1.05) | 0.16 |
| **Alcohol consumption^a^** |  |  |  |
| Non-current drinkers | 924 | Refence |  |
| 0-19 g of ethanol per day | 2019 | 0.79 (0.57, 1.09) | 0.16 |
| 20-39 g of ethanol per day | 1307 | 0.81 (0.58, 1.15) | 0.24 |
| ≥40 g of ethanol per day | 1510 | 0.86 (0.61, 1.19) | 0.36 |
| **Diabetes status** |  |  |  |
| No | 5110 | Reference |  |
| Yes | 663 | 1.52 (1.16, 1.99) | 0.003 |
| **Employment at Tokyo Electric Power Company in 2011** |  |  |  |
| No | 3568 | Reference |  |
| Yes | 2205 | 0.93 (0.70, 1.23) | 0.60 |

^a^ Hazard ratios for alcohol consumption were obtained from a model using the four-category classification instead of the binary variable; all other covariates were estimated from the model including alcohol consumption as a binary variable.

^a^ Alcohol consumption was assessed using a beverage-specific questionnaire that collected information on drinking frequency and usual amount consumed per occasion for each type of alcoholic beverage. Average daily alcohol intake was calculated by multiplying the frequency of consumption by the amount consumed per occasion and the ethanol content of each beverage, summing across all beverage types, and dividing by seven to obtain grams of ethanol per day.

Supplementary Table S2 Cumulative occupational radiation exposure up to November 2011 and risk of cataract, stratified by selected covariates

| Covariates | N | Hazard ratio Per 10 mSv (95% CI) | P for interaction |
| --- | --- | --- | --- |
| **Age in 2011 (years)** |  |  |  |
| <45 | 2538 | 1.08 (1.04, 1.12) |  |
| ≥45 | 3235 | 1.03 (1.01, 1.05) | 0.16 |
| **Body mass index (kg/m2)** |  |  |  |
| <25 | 3537 | 1.04 (1.02, 1.06) |  |
| ≥25 | 2236 | 1.03 (1.00, 1.05) | 0.89 |
| **Current smokers** |  |  |  |
| No | 3817 | 1.03 (1.01, 1.05) |  |
| Yes | 1956 | 1.04 (1.01, 1.06) | 0.57 |
| **Curren drinkers** |  |  |  |
| No | 924 | 1.02 (0.99, 1.06) |  |
| Yes | 4849 | 1.04 (1.02, 1.06) | 0.43 |
| **Alcohol consumption^a^** |  |  |  |
| Non-current drinkers | 924 | 1.02 (0.99, 1.06) |  |
| 0-19 g of ethanol per day | 2019 | 1.07 (1.04, 1.10) |  |
| 20-39 g of ethanol per day | 1307 | 1.03 (0.99, 1.06) |  |
| ≥40 g of ethanol per day | 1510 | 1.01 (0.97, 1.05) | 0.51 |
| **Diabetes status** |  |  |  |
| No | 5110 | 1.03 (1.01, 1.05) |  |
| Yes | 663 | 1.04 (1.01, 1.07) | 0.83 |
| **Employment at Tokyo Electric Power Company in 2011** |  |  |  |
| No | 3568 | 1.03 (1.01, 1.05) |  |
| Yes | 2205 | 1.05 (1.02, 1.08) | 0.35 |

^a^ For the alternative alcohol-stratified analyses, alcohol consumption was categorized as non-current drinkers, 0–19 g/day, 20–39 g/day, and ≥40 g/day; for all other stratified analyses, alcohol consumption was included in the model as a binary variable (non-current drinker vs. current drinker).
